# Supplementary material for: BRACNAC: A BRCA1 and BRCA2 Copy Number Alteration Caller from Next-Generation Sequencing Data
Source: Int J Mol Sci. 2023 Nov 22;24(23):16630. doi: 10.3390/ijms242316630 (PMC10706169; doi:10.3390/ijms242316630)
Supplement: Supplementary file 1 [file ijms-24-16630-s001.zip › Supplementary Figures.pdf]

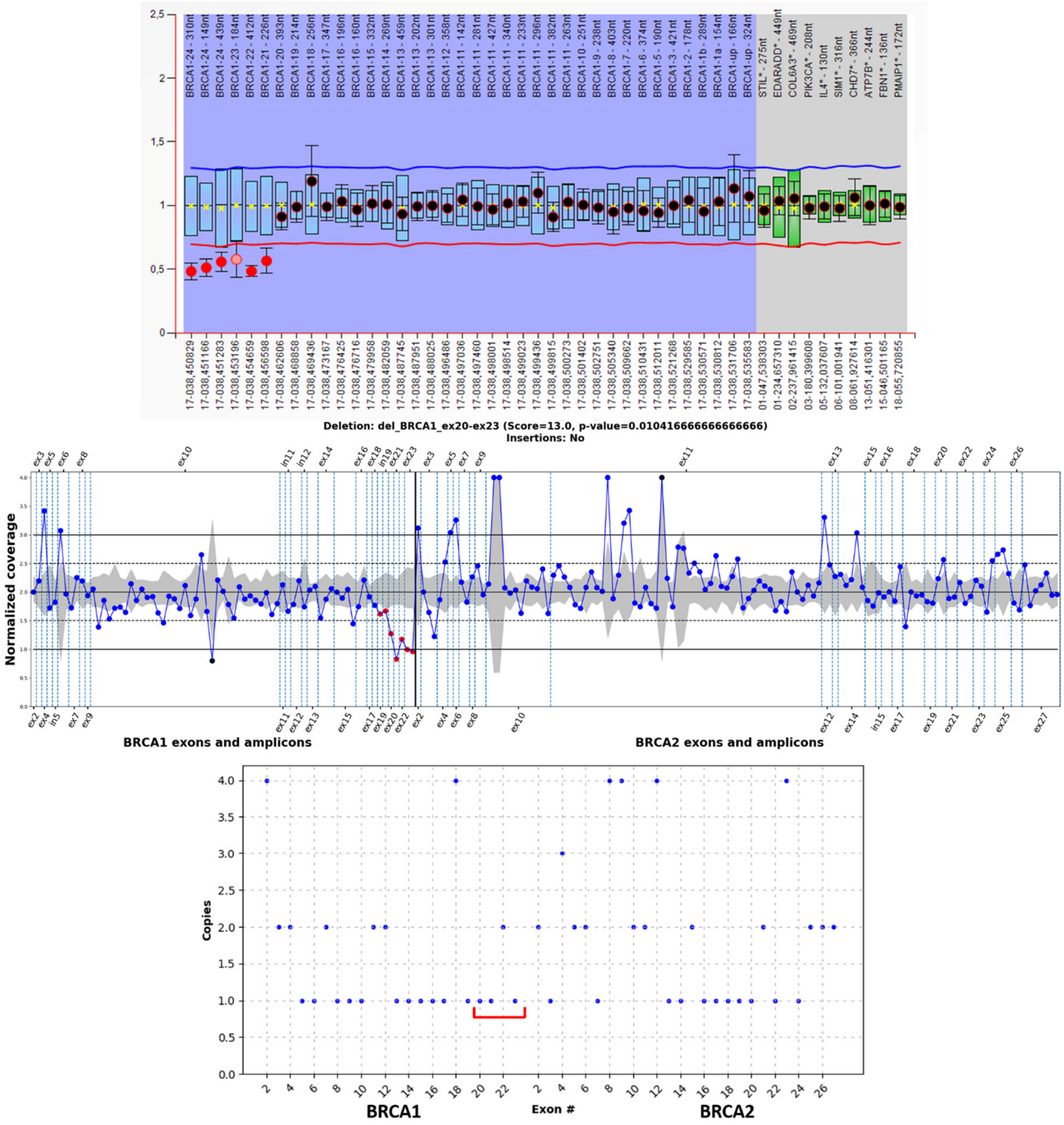

Figure S1. Ratio plots for mlpa_1 DNA sample from MLPA (upper), BRACNAC using the best parameters (middle), and panelcn.MOPS (lower). For BRACNAC, all target regions potentially affected by CNV are highlighted in red, but the final result is written in the figure top. For panelcn.MOPS, exons affected are underlined with the red bracket. This sample has the deletion of exons 20–23 of the *BRCA1* gene.

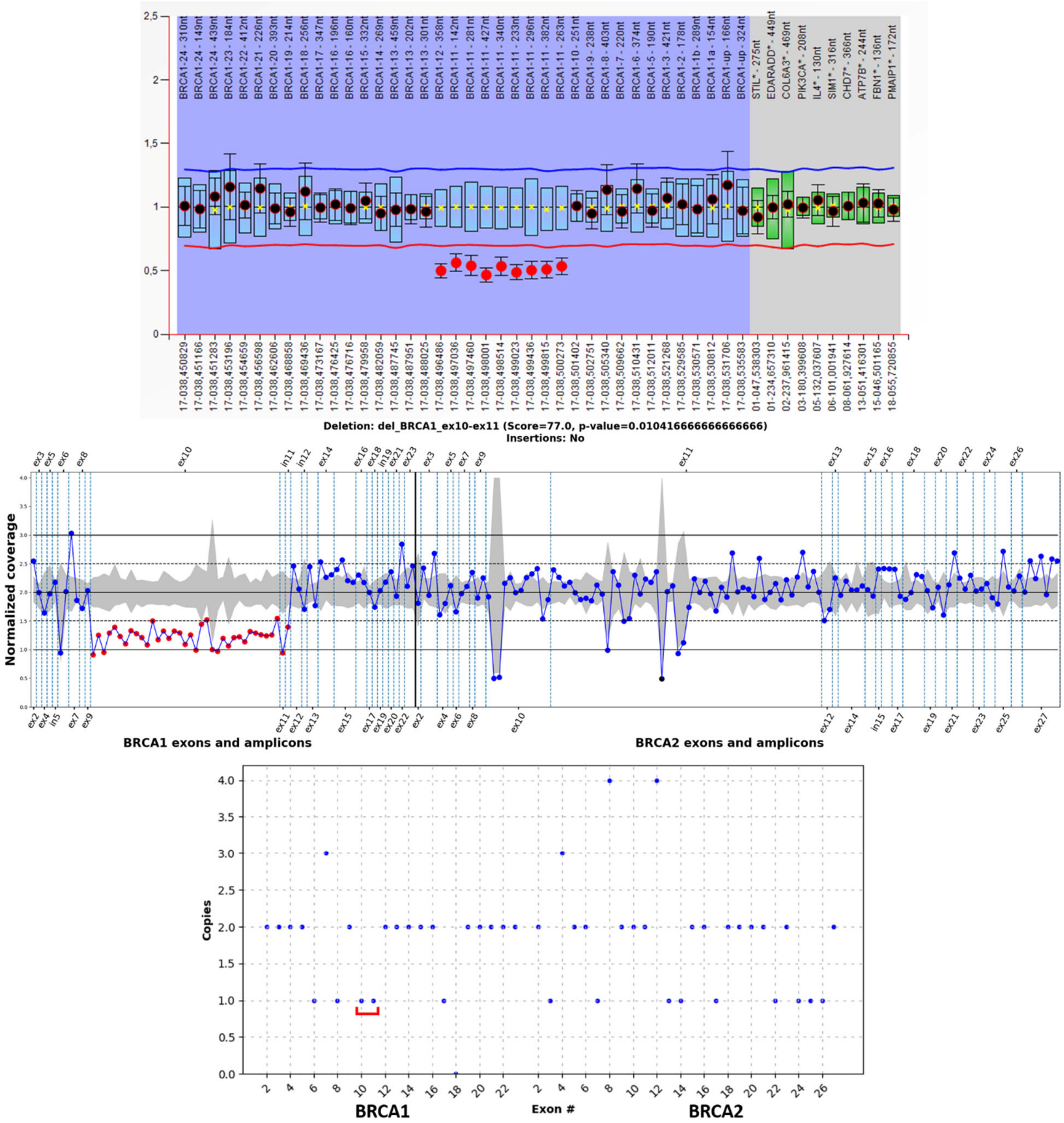

Figure S2. Ratio plots for mlpa_2 DNA sample from MLPA (upper), BRACNAC using the best parameters (middle), and panelcn.MOPS (lower). For BRACNAC, all target regions potentially affected by CNV are highlighted in red, but the final result is written in the figure top.

For panelcn.MOPS, exons affected are underlined with the red bracket. This sample has the deletion of exons 10–11 of the *BRCA1* gene.

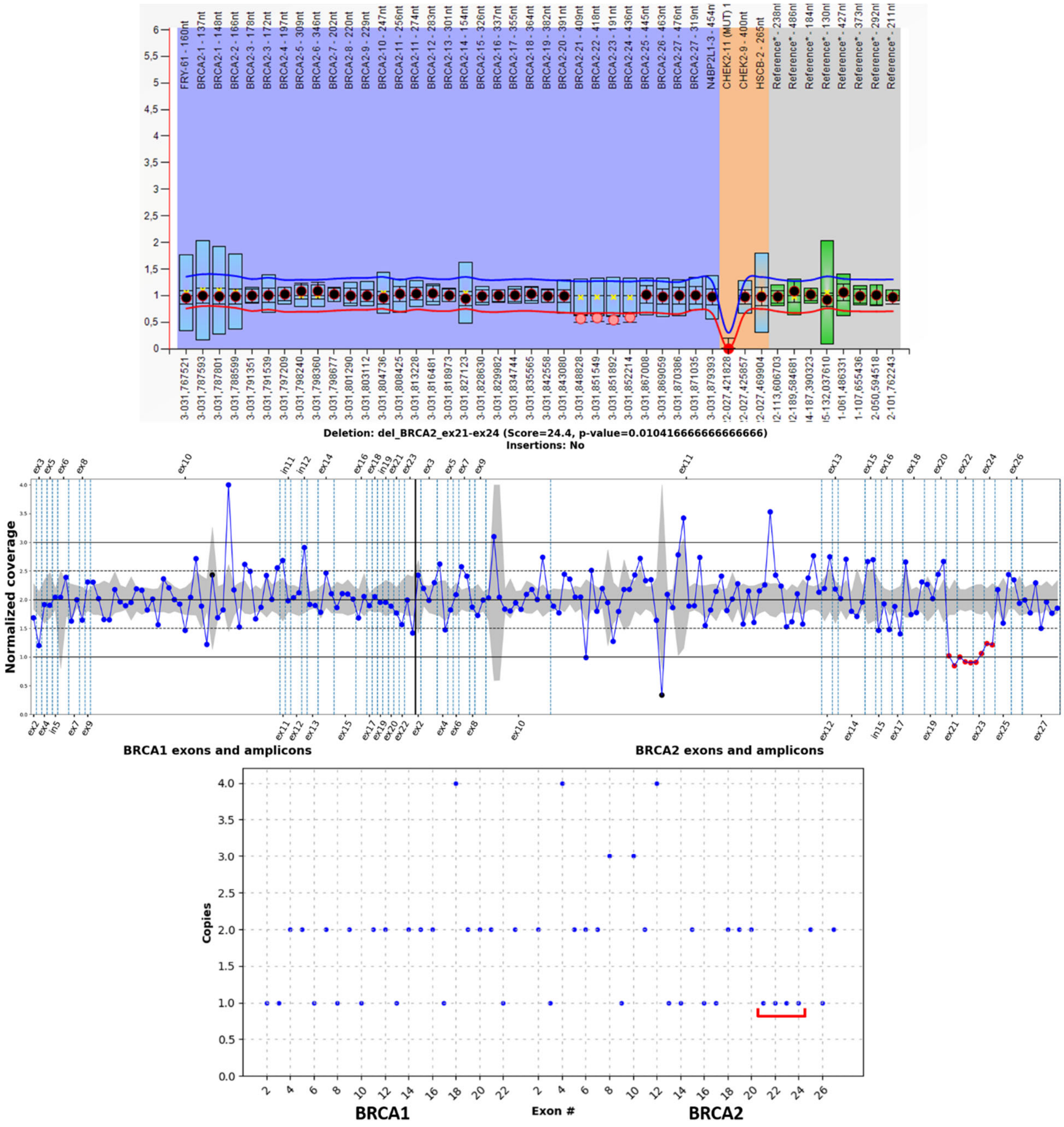

Figure S3. Ratio plots for mlpa_3 DNA sample from MLPA (upper), BRACNAC using the best parameters (middle), and panelcn.MOPS (lower). For BRACNAC, all target regions potentially affected by CNV are highlighted in red, but the final result is written in the figure top. For panelcn.MOPS, exons affected are underlined with the red bracket. This sample has the deletion of exons 21–24 of the *BRCA2* gene.

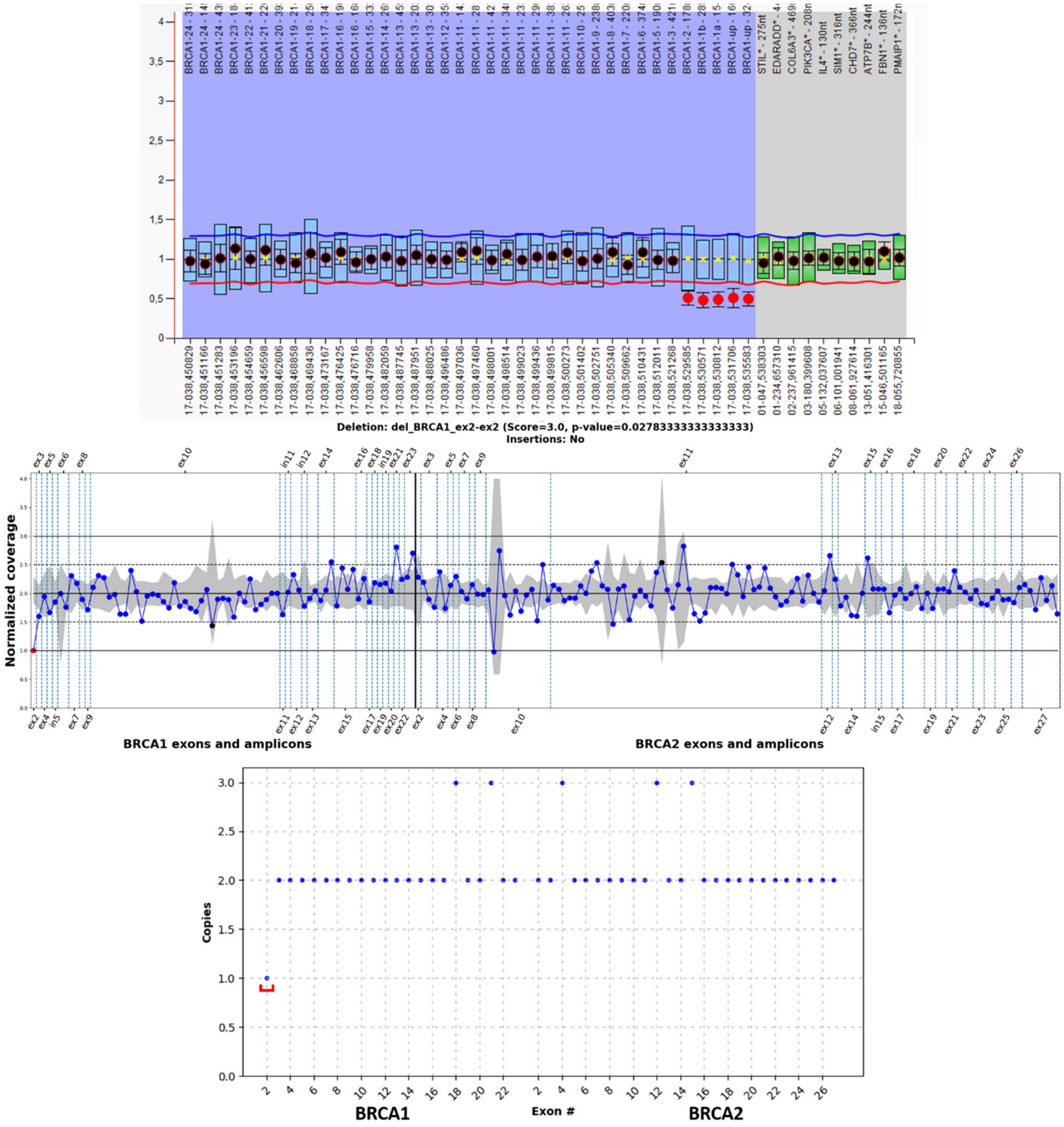

Figure S4. Ratio plots for mlpa_4 DNA sample from MLPA (upper), BRACNAC using the best parameters (middle), and panelcn.MOPS (lower). For BRACNAC, all target regions potentially affected by CNV are highlighted in red, but the final result is written in the figure top. For panelcn.MOPS, exons affected are underlined with the red bracket. This sample has the deletion of promotor and exons 1–2 of the *BRCA1* gene.

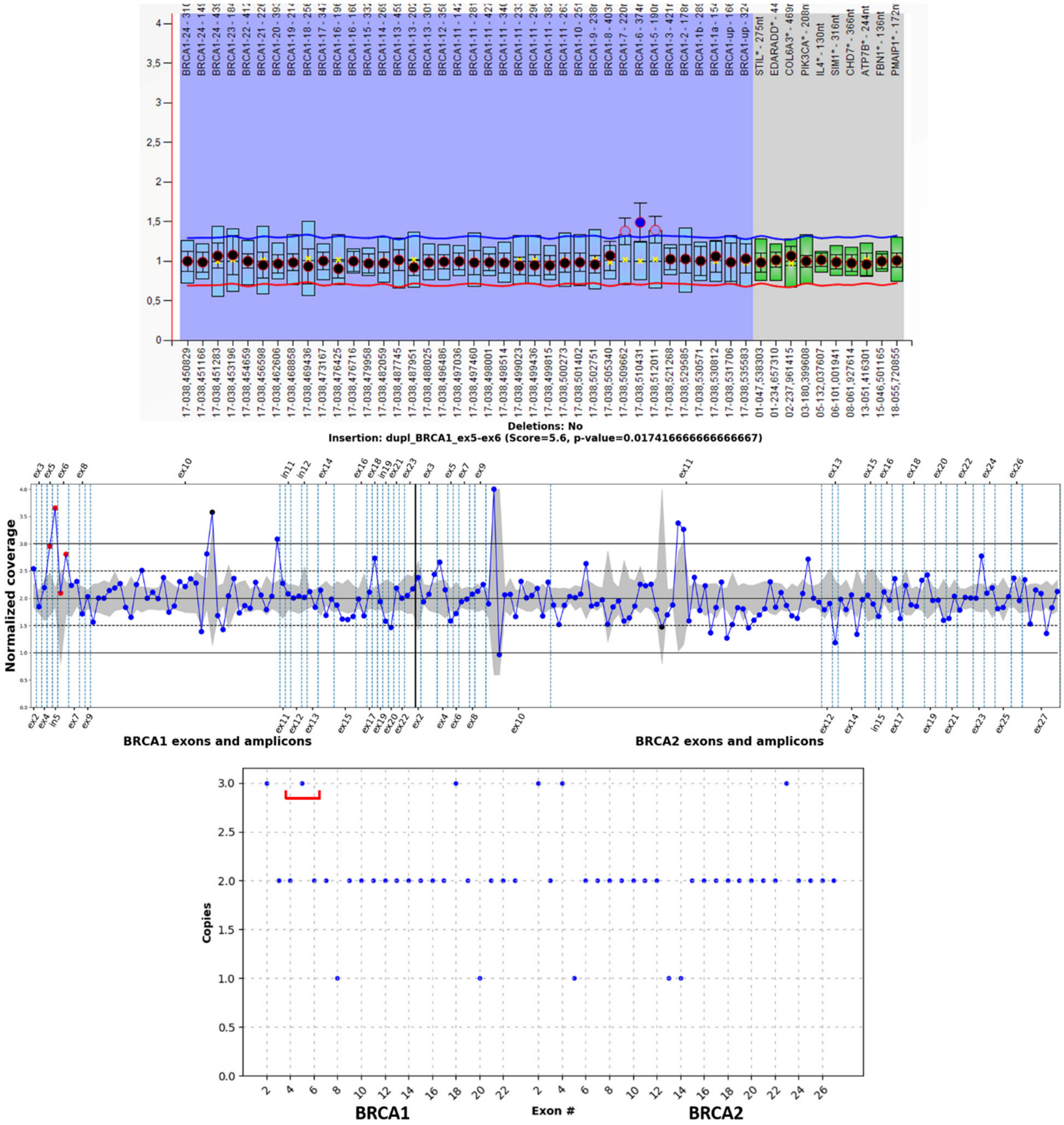

Figure S5. Ratio plots for mlpa_5 DNA sample from MLPA (upper), BRACNAC using the best parameters (middle), and panelcn.MOPS (lower). For BRACNAC, all target regions potentially affected by CNV are highlighted in red, but the final result is written in the figure top. For panelcn.MOPS, exons affected are underlined with the red bracket. This sample has the duplication of exons 4–6 of the *BRCA1* gene.

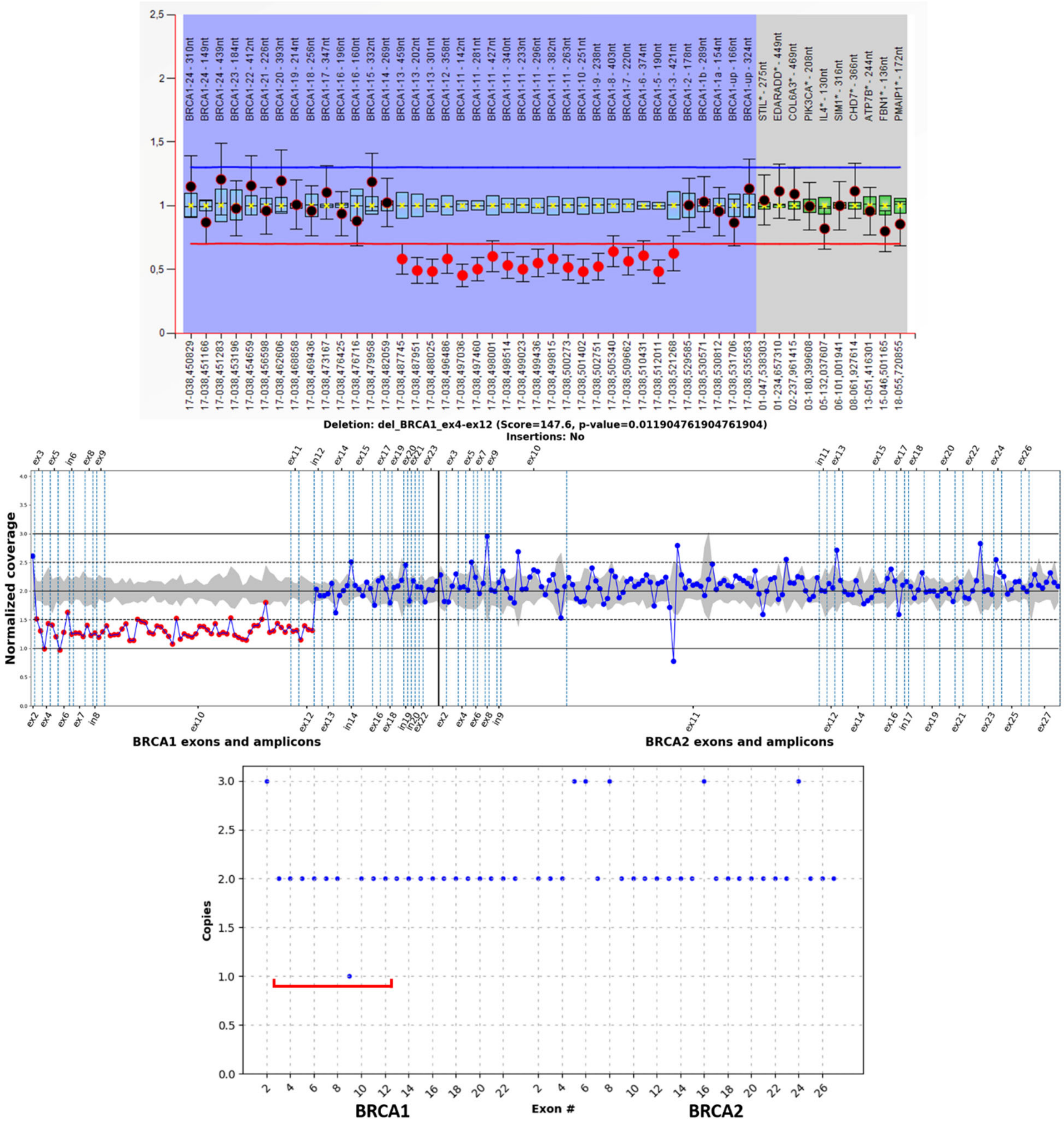

Figure S6. Ratio plots for mlpa_6 DNA sample from MLPA (upper), BRACNAC using the best parameters (middle), and panelcn.MOPS (lower). For BRACNAC, all target regions potentially affected by CNV are highlighted in red, but the final result is written in the figure top. For panelcn.MOPS, exons affected are underlined with the red bracket. This sample has the deletion of exons 3–12 of the *BRCA1* gene.

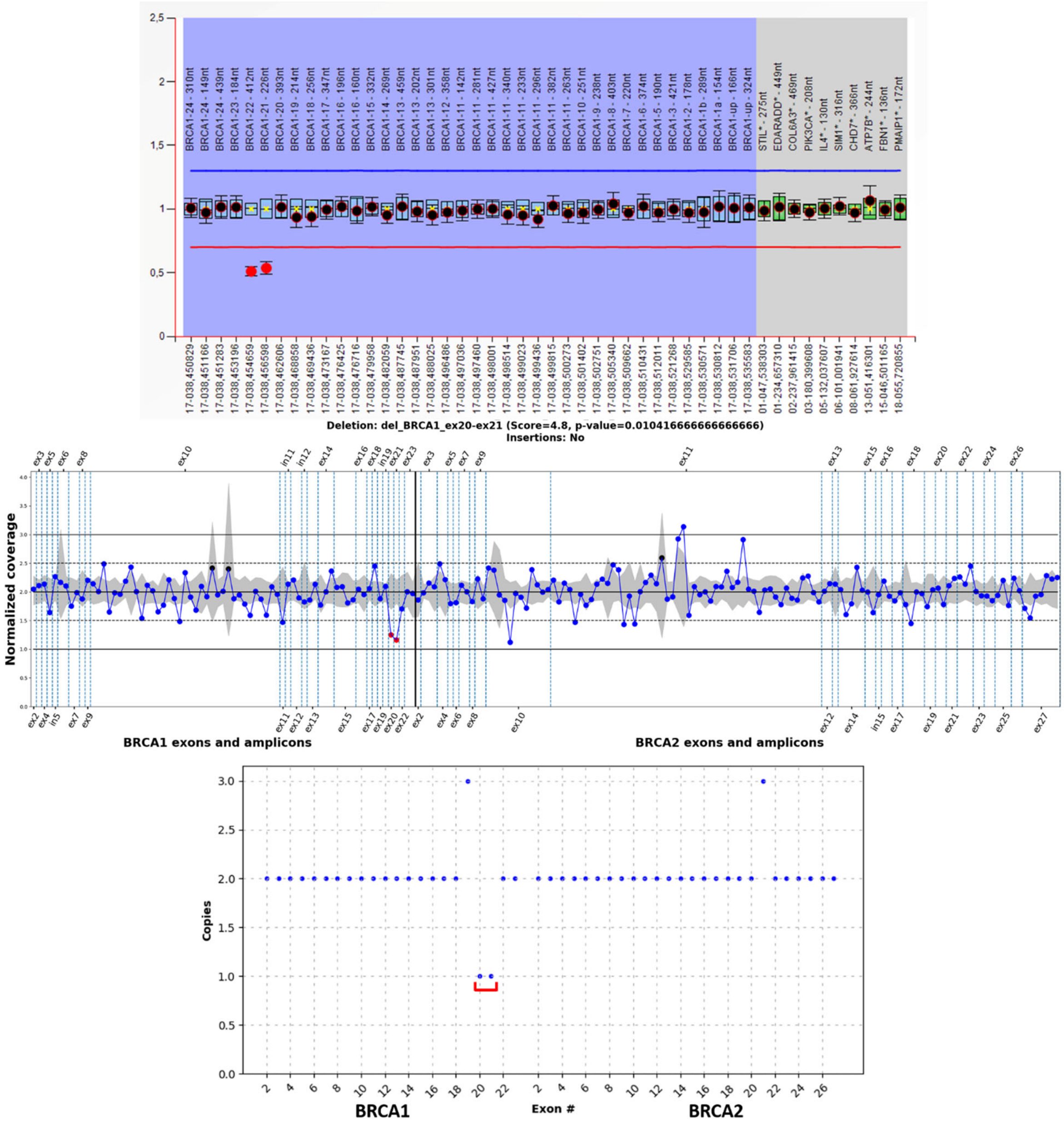

Figure S7. Ratio plots for mlpa_7 DNA sample from MLPA (upper), BRACNAC using the best parameters (middle), and panelcn.MOPS (lower). For BRACNAC, all target regions potentially affected by CNV are highlighted in red, but the final result is written in the figure top. For panelcn.MOPS, exons affected are underlined with the red bracket. This sample has the deletion of exons 20–21 of the *BRCA1* gene.

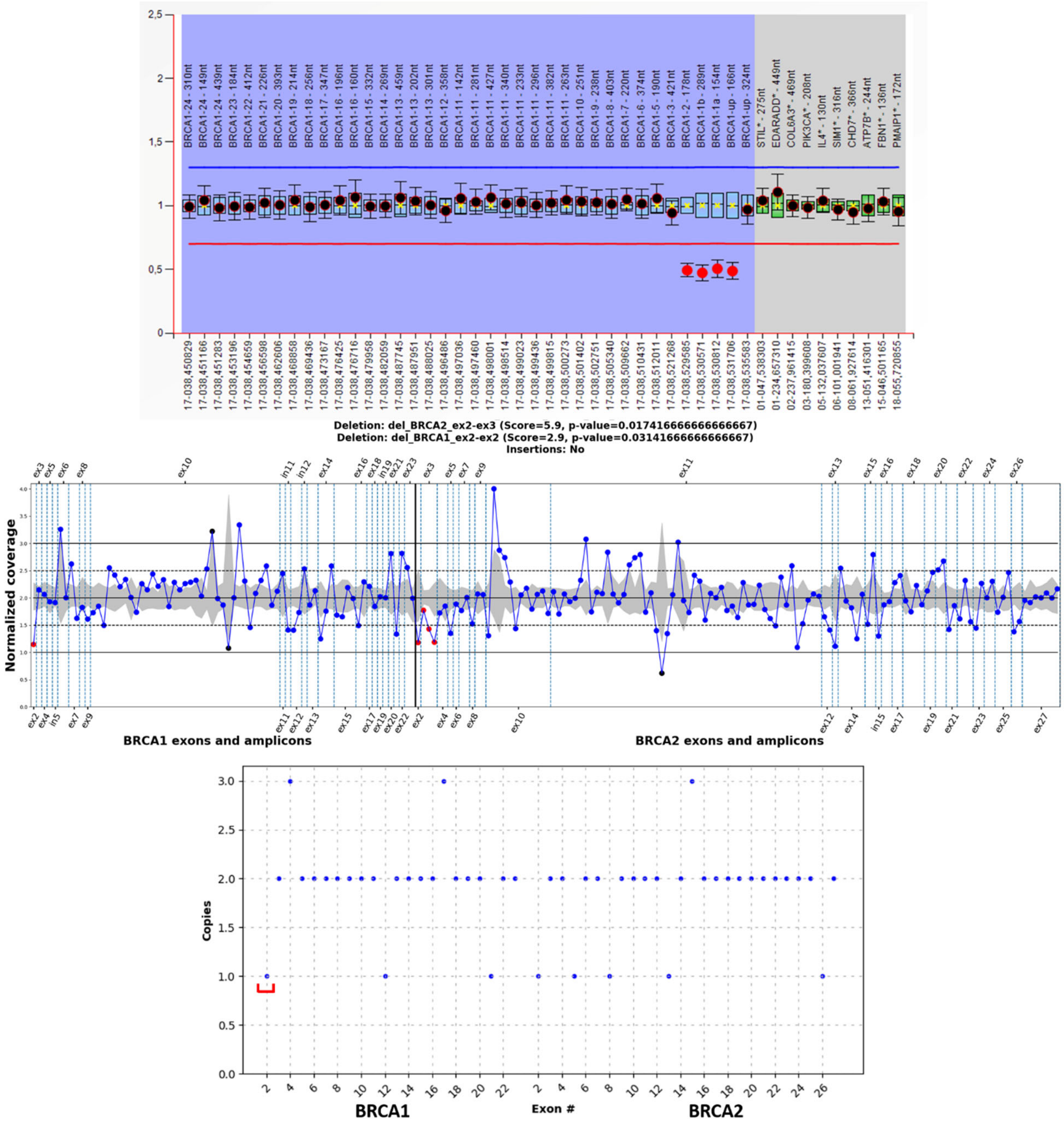

Figure S8. Ratio plots for mlpa_8 DNA sample from MLPA (upper), BRACNAC using the best parameters (middle), and panelcn.MOPS (lower). For BRACNAC, all target regions potentially affected by CNV are highlighted in red, but the final result is written in the figure top. For panelcn.MOPS, exons affected are underlined with the red bracket. This sample has the deletion of promotor and exons 1–2 of the *BRCA1* gene.

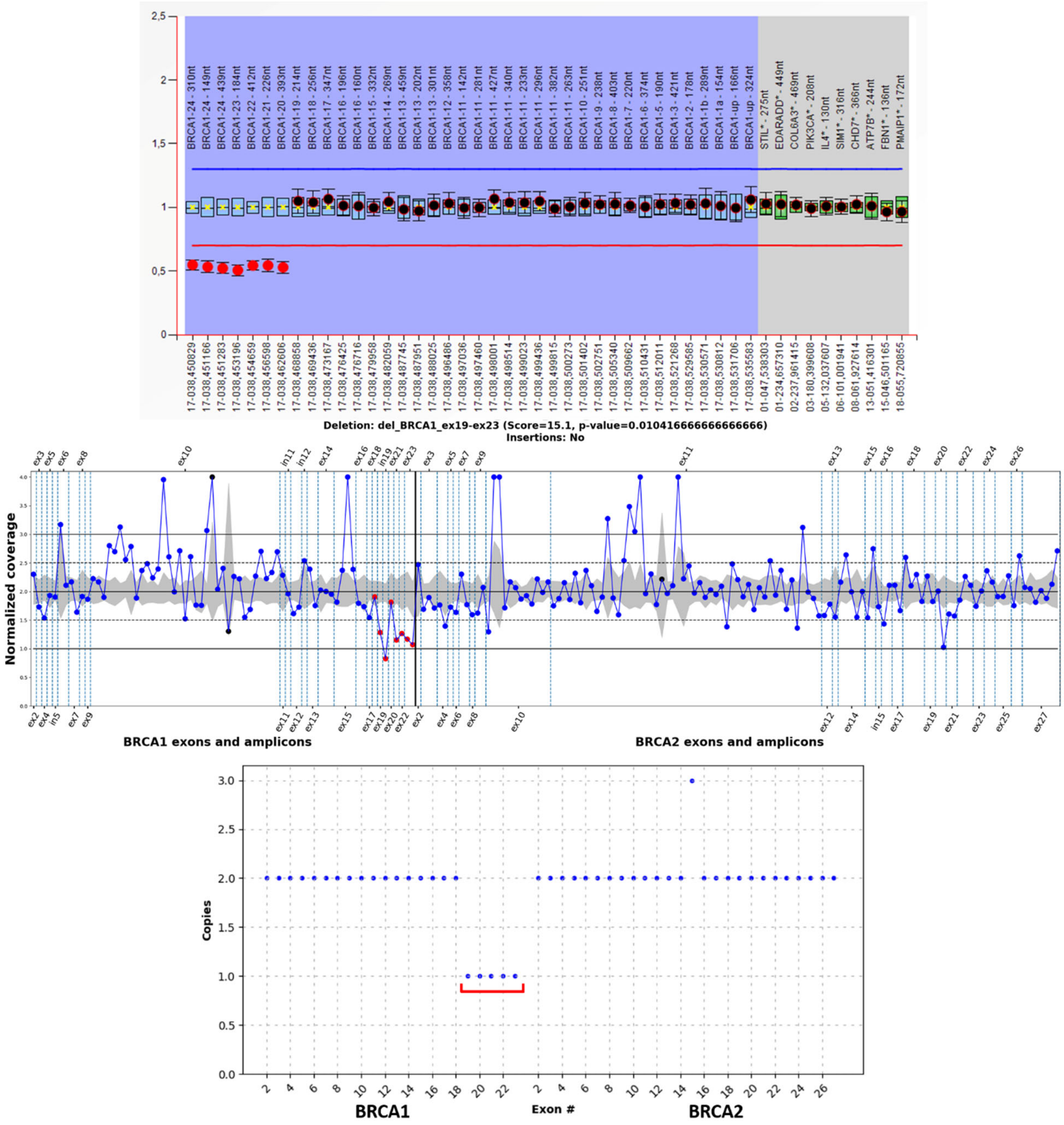

Figure S9. Ratio plots for mlpa_9 DNA sample from MLPA (upper), BRACNAC using the best parameters (middle), and panelcn.MOPS (lower). For BRACNAC, all target regions potentially affected by CNV are highlighted in red, but the final result is written in the figure top. For panelcn.MOPS, exons affected are underlined with the red bracket. This sample has the deletion of exons 19–23 of the *BRCA1* gene.

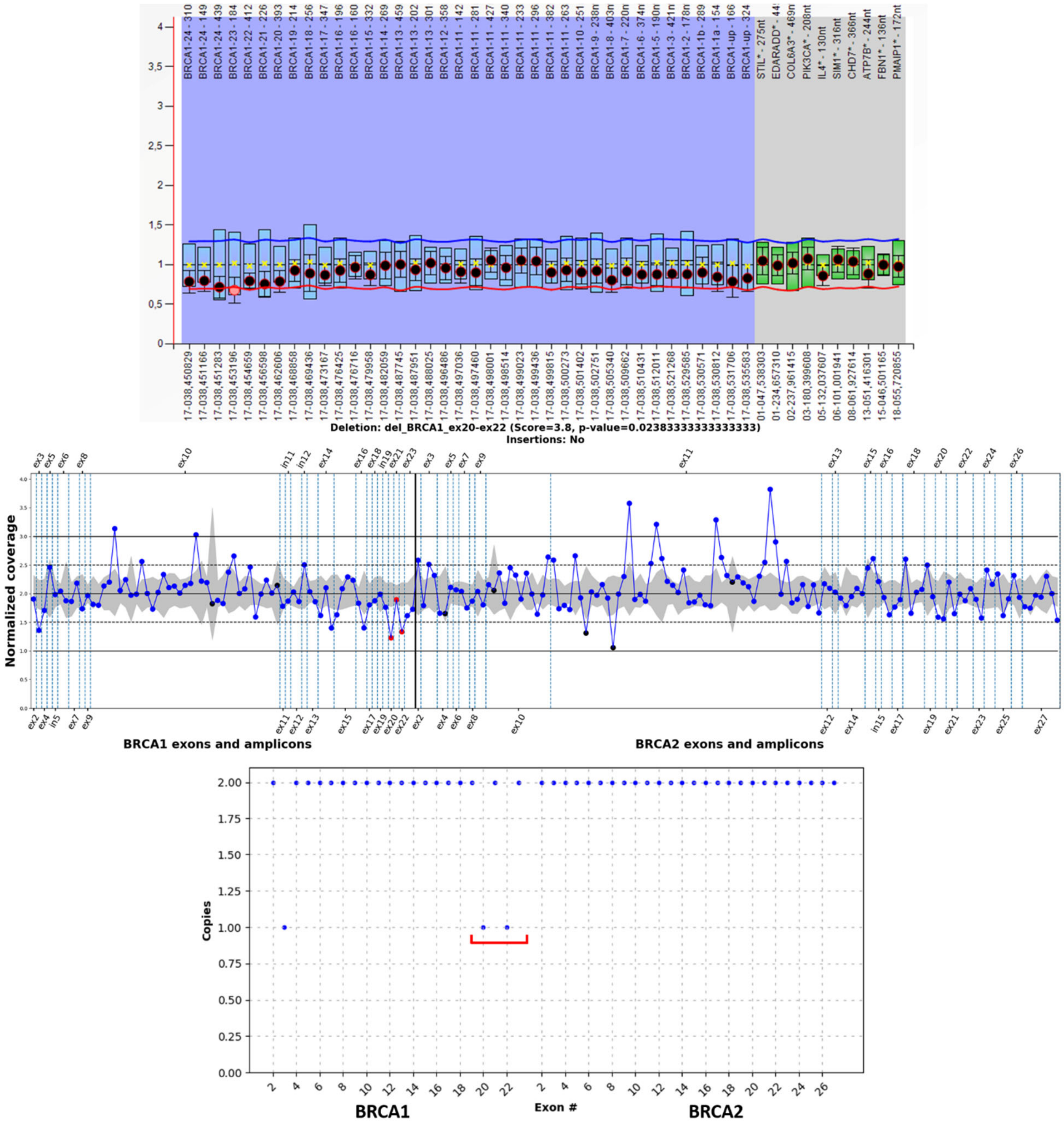

Figure S10. Ratio plots for mlpa_10 DNA sample from MLPA (upper), BRACNAC using the best parameters (middle), and panelcn.MOPS (lower). For BRACNAC, all target regions potentially affected by CNV are highlighted in red, but the final result is written in the figure top. For panelcn.MOPS, exons affected are underlined with the red bracket. This sample has the deletion of exons 19–23 of the *BRCA1* gene.

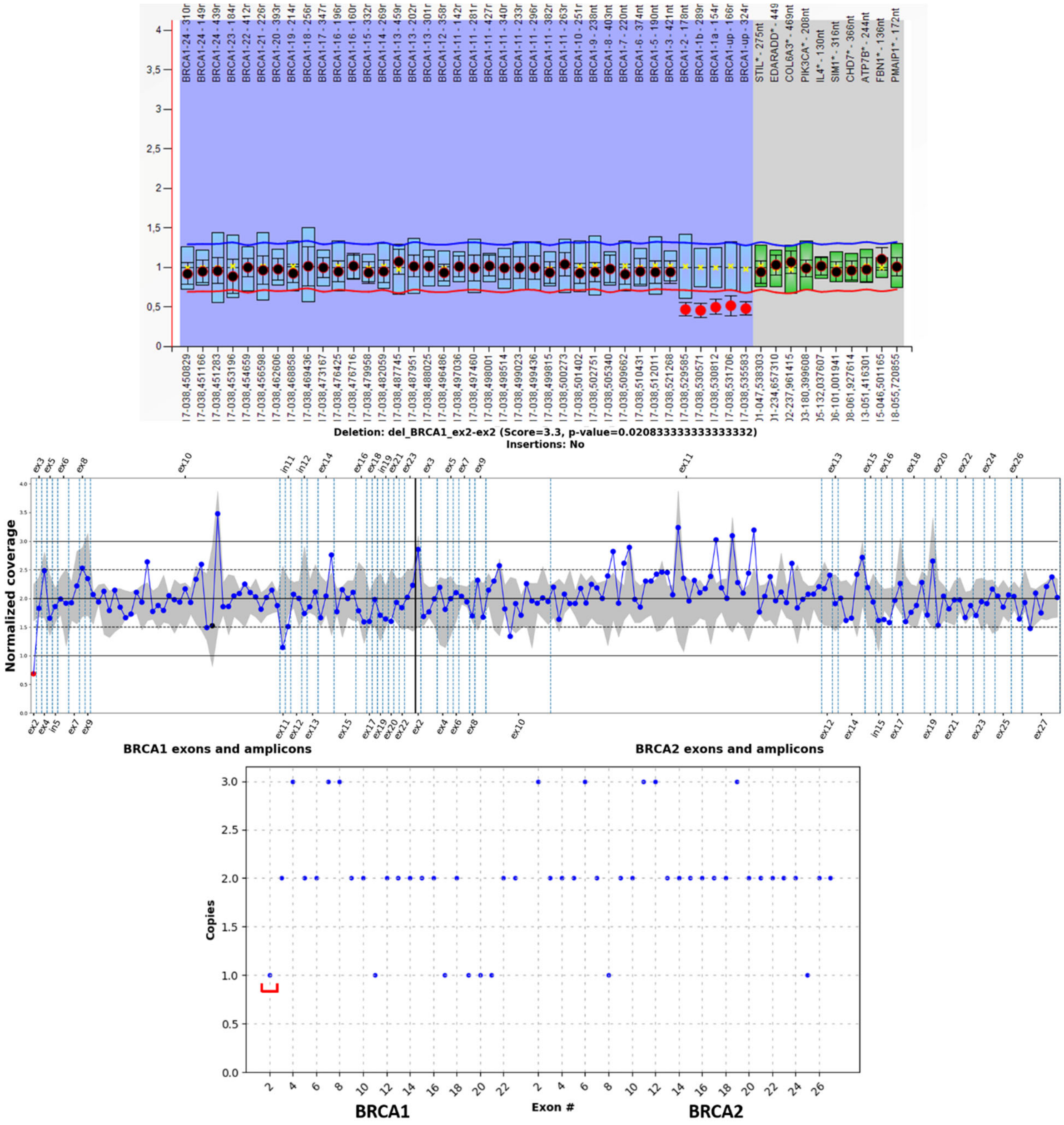

Figure S11. Ratio plots for mlpa_11 DNA sample from MLPA (upper), BRACNAC using the best parameters (middle), and panelcn.MOPS (lower). For BRACNAC, all target regions potentially affected by CNV are highlighted in red, but the final result is written in the figure top. For panelcn.MOPS, exons affected are underlined with the red bracket. This sample has the deletion of promotor and exons 1–2 of the *BRCA1* gene.

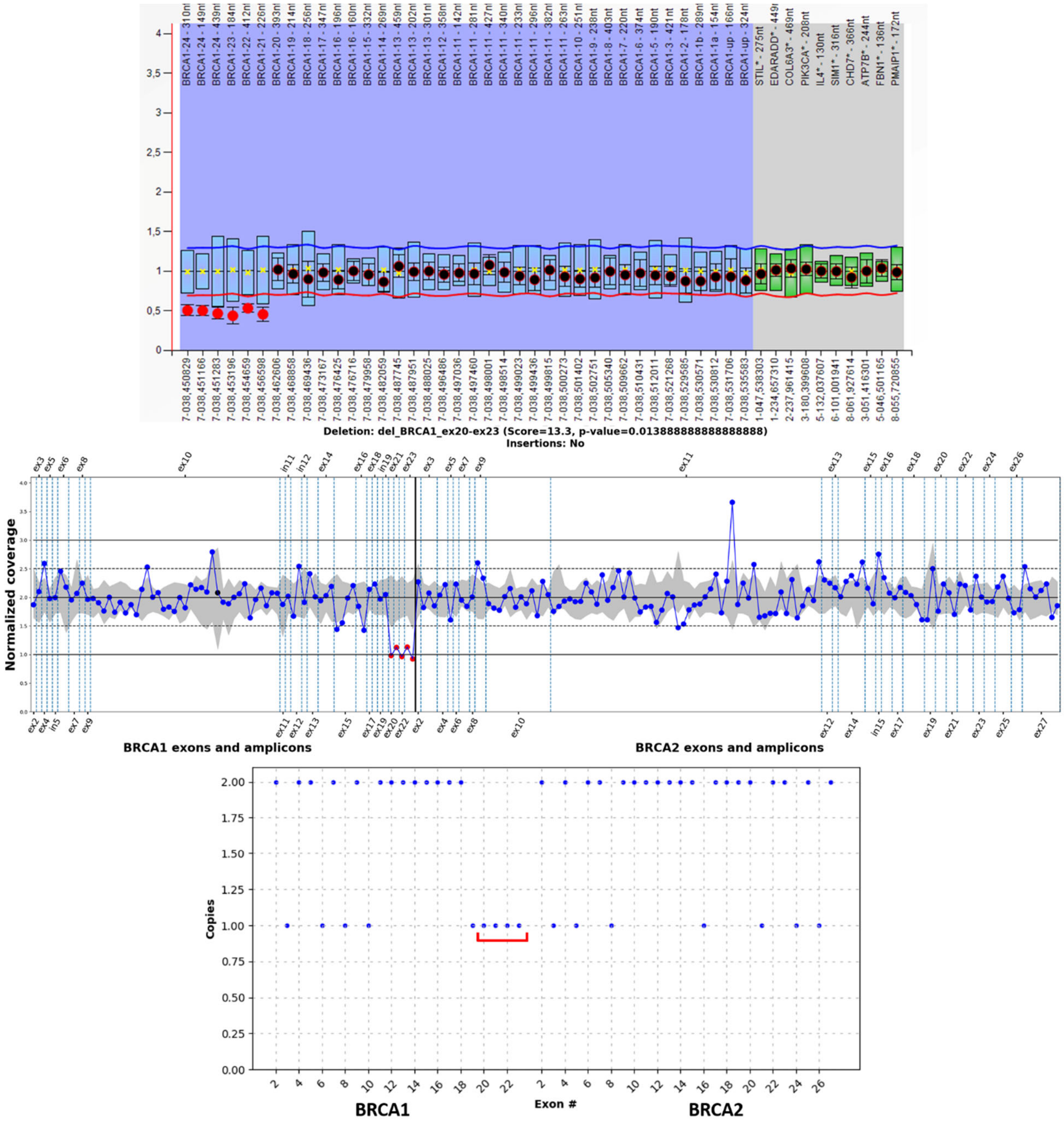

Figure S12. Ratio plots for mlpa_12 DNA sample from MLPA (upper), BRACNAC using the best parameters (middle), and panelcn.MOPS (lower). For BRACNAC, all target regions potentially affected by CNV are highlighted in red, but the final result is written in the figure top. For panelcn.MOPS, exons affected are underlined with the red bracket. This sample has the deletion of exons 20–23 of the *BRCA1* gene.

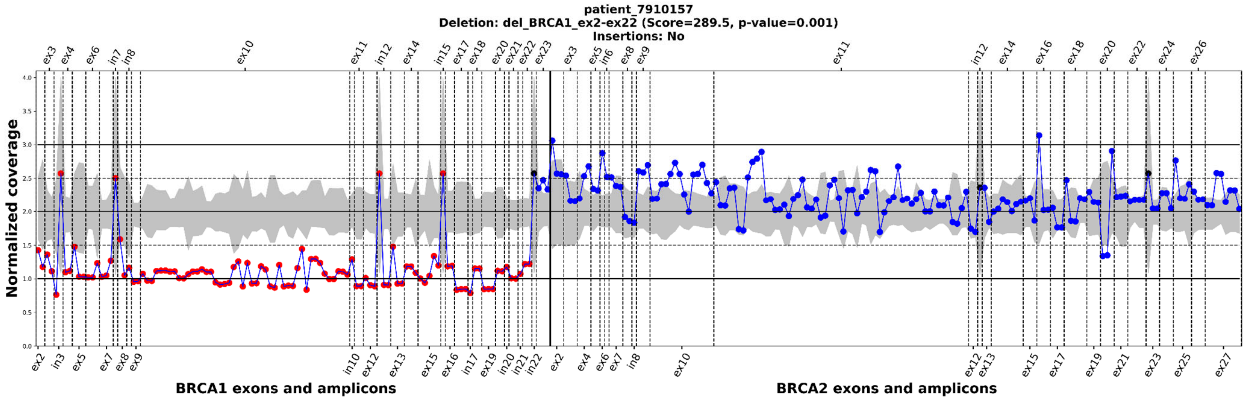

Figure S13. Ratio plots for SRR7910157. All target regions potentially affected by CNV are highlighted in red, but the final result is written in the figure top. Here it is *BRCA1* exons 2–22 deletion.

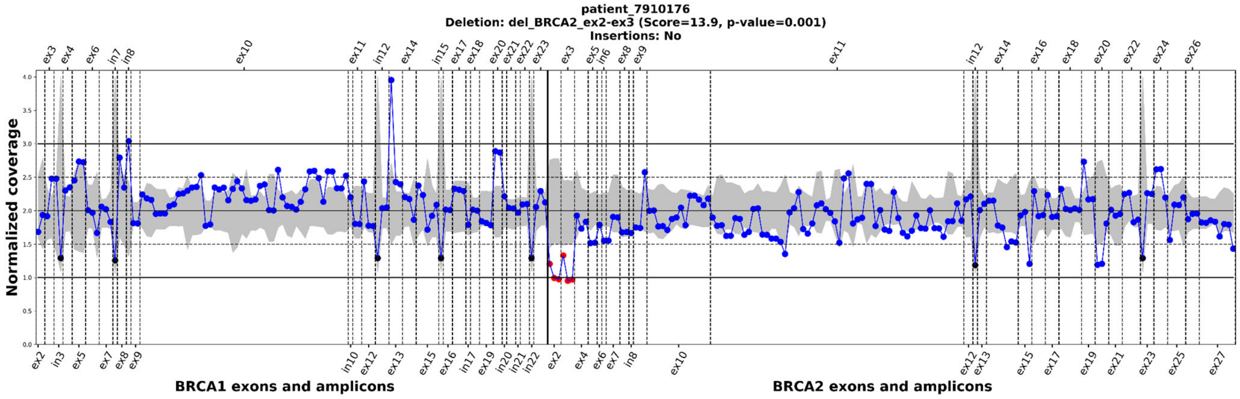

Figure S14. Ratio plots for SRR7910176. All target regions potentially affected by CNV are highlighted in red, but the final result is written in the figure top. Here it is *BRCA2* exons 2–3 deletion.

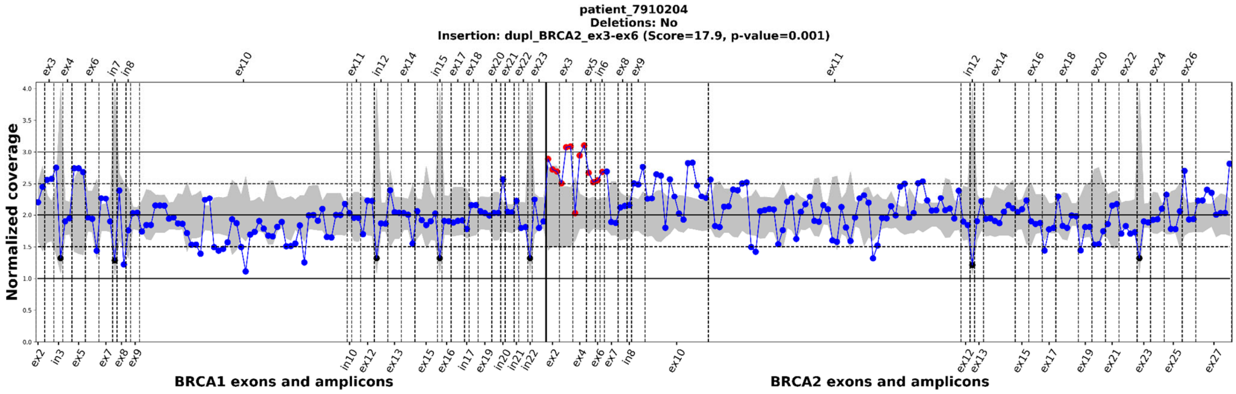

Figure S15. Ratio plots for SRR7910204. All target regions potentially affected by CNV are highlighted in red, but the final result is written in the figure top. Here it is *BRCA2* exons 3–6 duplication.

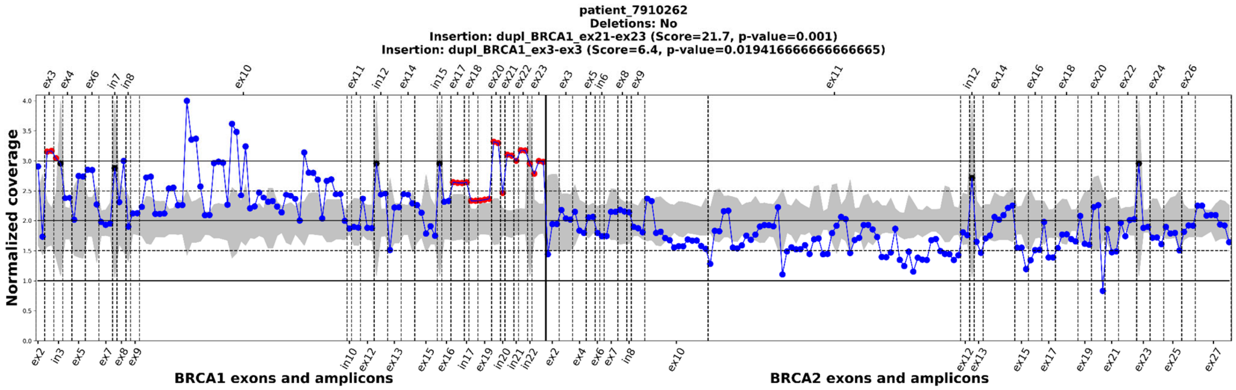

Figure S16. Ratio plots for SRR7910262. All target regions potentially affected by CNV are highlighted in red, but the final result is written in the figure top. Here it is *BRCA1* exons 21–23 duplication.

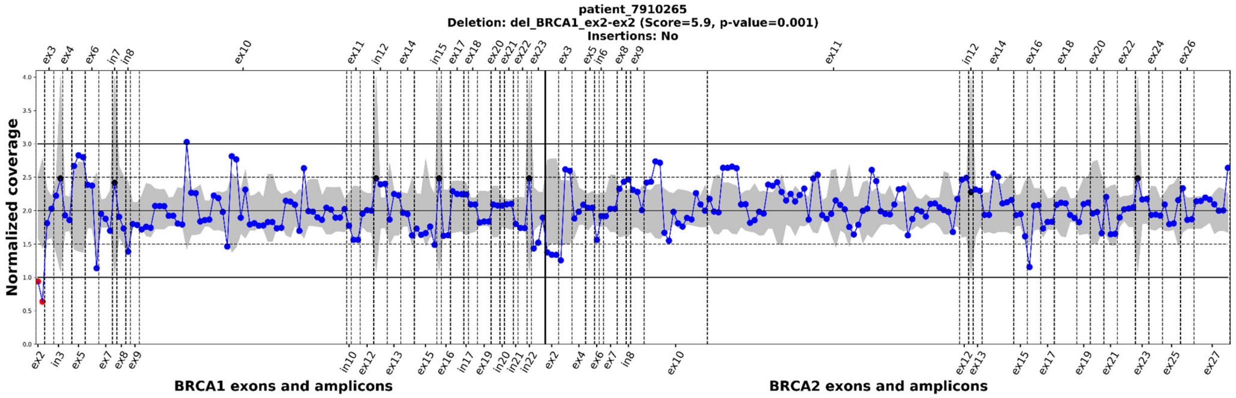

Figure S17. Ratio plots for SRR7910265. All target regions potentially affected by CNV are highlighted in red, but the final result is written in the figure top. Here it is *BRCA1* exon 2 deletion.

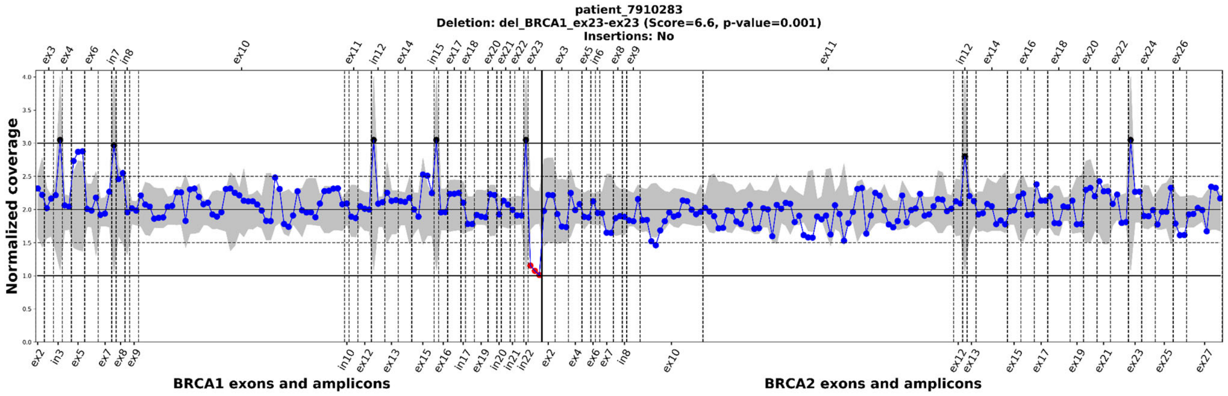

Figure S18. Ratio plots for SRR7910283. All target regions potentially affected by CNV are highlighted in red, but the final result is written in the figure top. Here it is *BRCA1* exon 23 deletion.

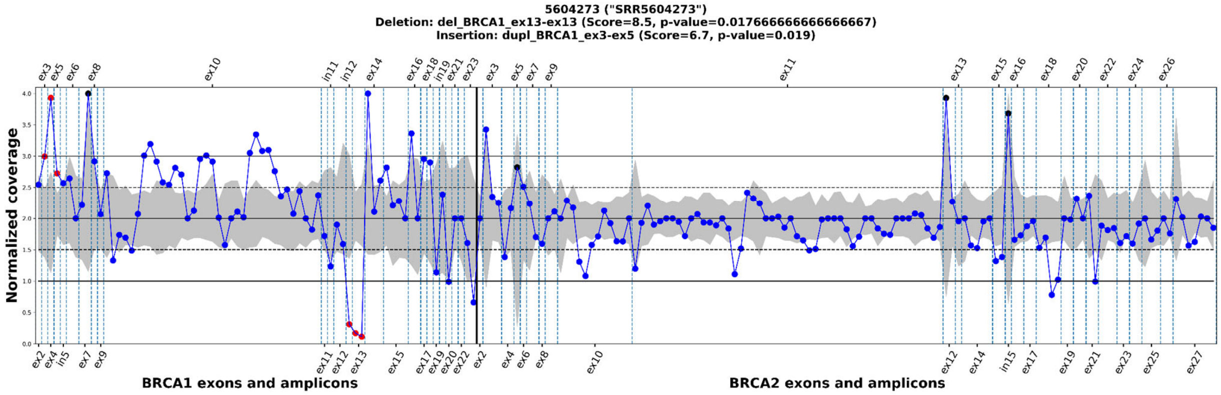

Figure S19. Ratio plots for SRR5604273. All target regions potentially affected by CNV are highlighted in red, but the final result is written in the figure top. Here it is *BRCA1* exon 13 deletion.

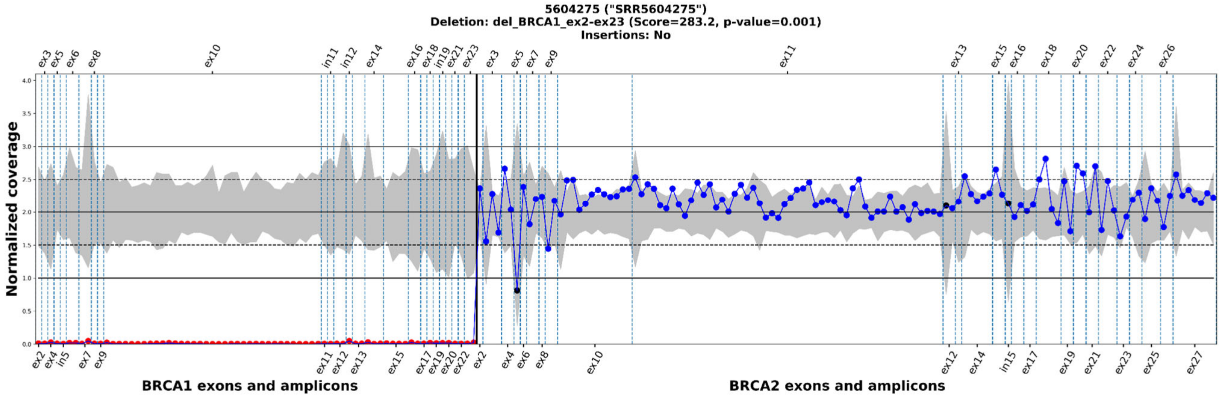

Figure S20. Ratio plots for SRR5604275. All target regions potentially affected by CNV are highlighted in red, but the final result is written in the figure top. Here it is *BRCA1* exons 2-23 deletion.

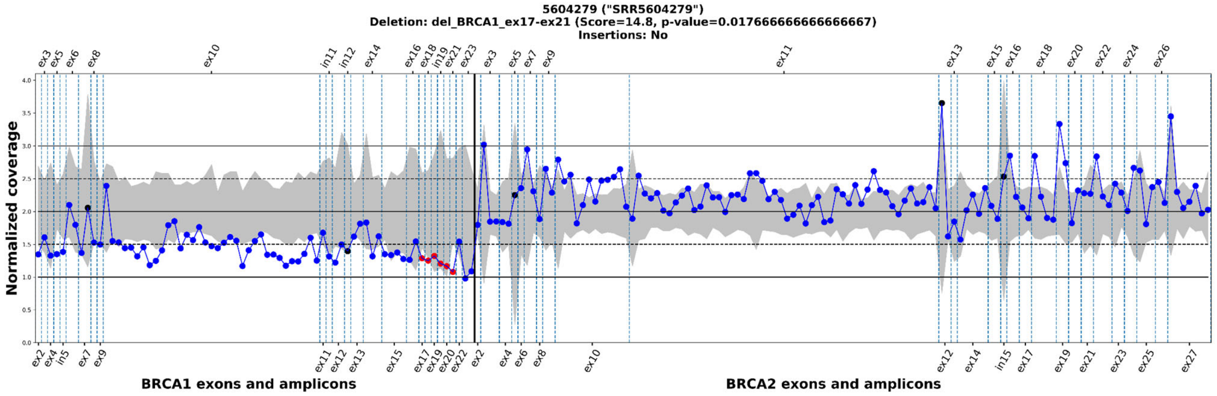

Figure S21. Ratio plots for SRR5604279. All target regions potentially affected by CNV are highlighted in red, but the final result is written in the figure top. Here it is *BRCA1* exons 17-21 deletion.

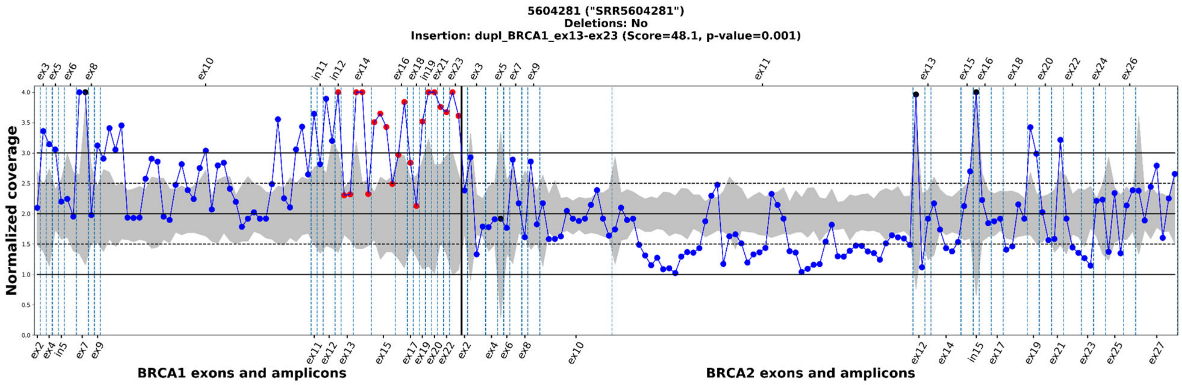

Figure S22. Ratio plots for SRR5604281. All target regions potentially affected by CNV are highlighted in red, but the final result is written in the figure top. Here it is *BRCA1* exons 15-23 duplication.

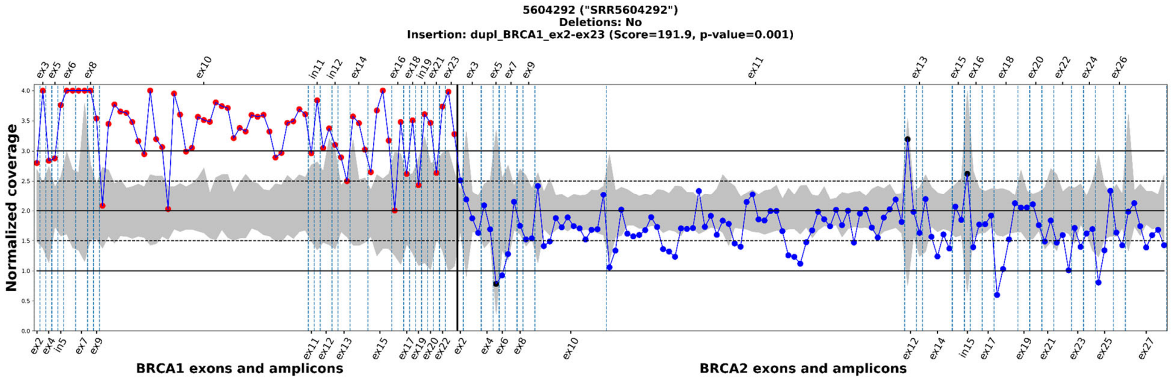

Figure S23. Ratio plots for SRR5604292. All target regions potentially affected by CNV are highlighted in red, but the final result is written in the figure top. Here it is *BRCA1* exons 2-23 duplication.

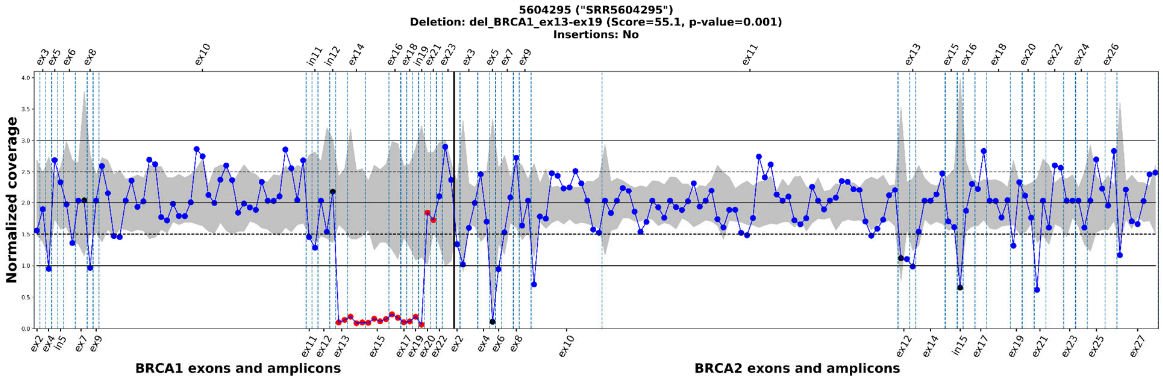

Figure S24. Ratio plots for SRR5604295. All target regions potentially affected by CNV are highlighted in red, but the final result is written in the figure top. Here it is *BRCA1* exons 13-19 deletion.

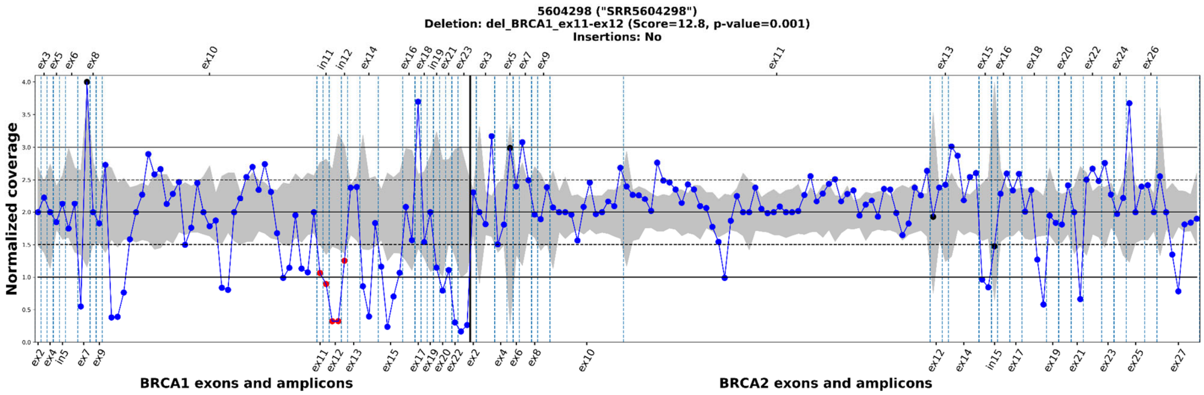

Figure S25. Ratio plots for SRR5604298. All target regions potentially affected by CNV are highlighted in red, but the final result is written in the figure top. Here it is *BRCA1* exons 11-12 deletion.

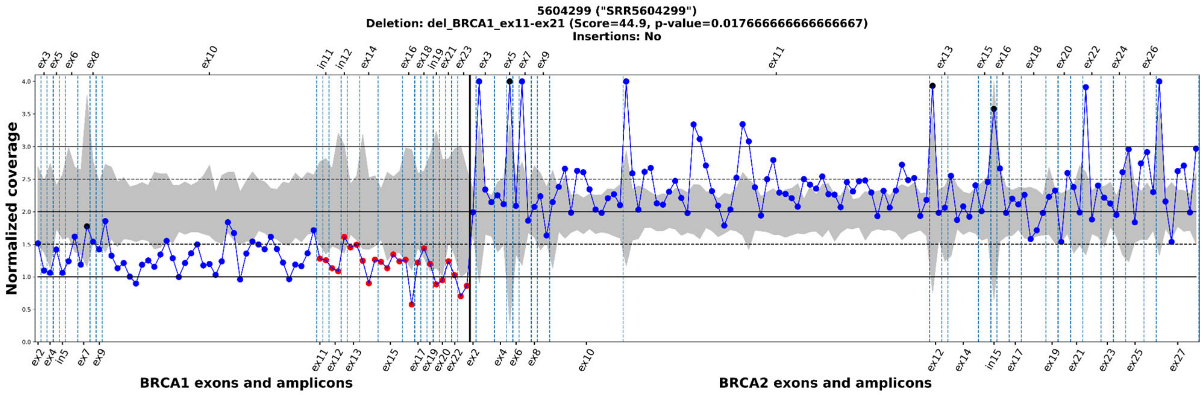

Figure S26. Ratio plots for SRR5604299. All target regions potentially affected by CNV are highlighted in red, but the final result is written in the figure top. Here it is *BRCA1* exons 11-21 deletion, however, whole *BRCA1* deletion is observed during manual analysis.

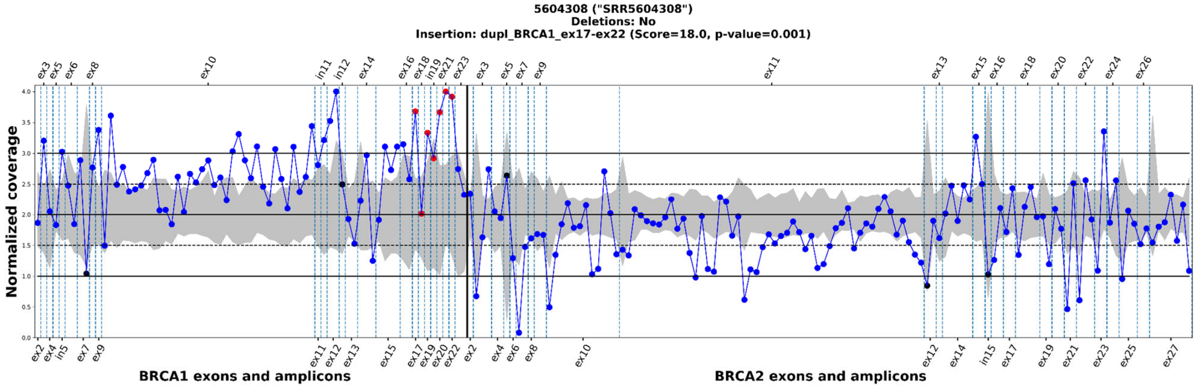

Figure S27. Ratio plots for SRR5604308. All target regions potentially affected by CNV are highlighted in red, but the final result is written in the figure top. Here it is *BRCA1* exons 17-22 duplication.

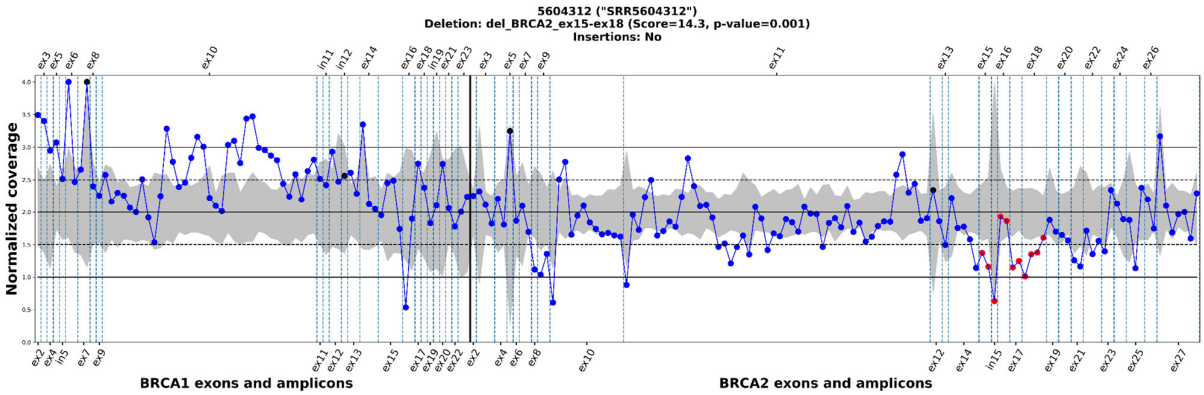

Figure S28. Ratio plots for SRR5604312. All target regions potentially affected by CNV are highlighted in red, but the final result is written in the figure top. Here it is *BRCA2* exons 15-18 deletion.

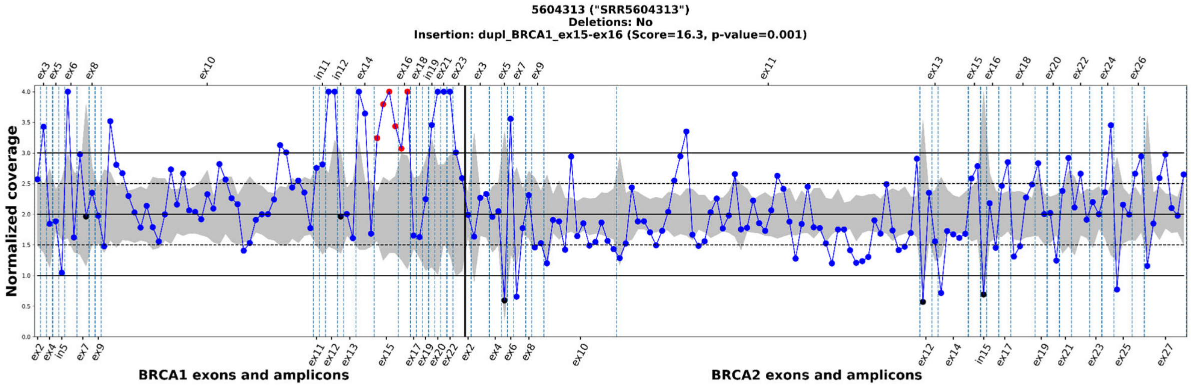

Figure S29. Ratio plots for SRR5604313. All target regions potentially affected by CNV are highlighted in red, but the final result is written in the figure top. Here it is *BRCA1* exons 15–16 duplication.

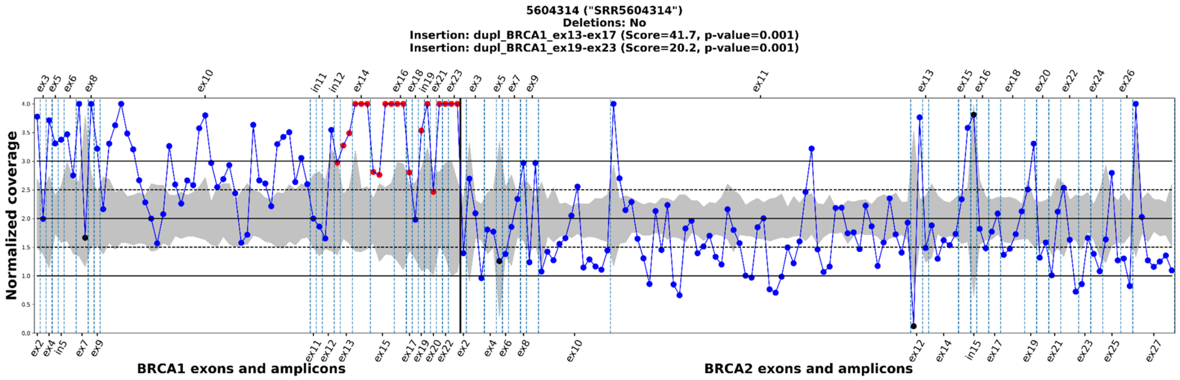

Figure S30. Ratio plots for SRR5604314. All target regions potentially affected by CNV are highlighted in red, but the final result is written in the figure top. Here it is *BRCA1* exons 13–17 and exons 19–23 duplications that can likely be joined into one duplication event *BRCA1* exons 13–23 duplication.

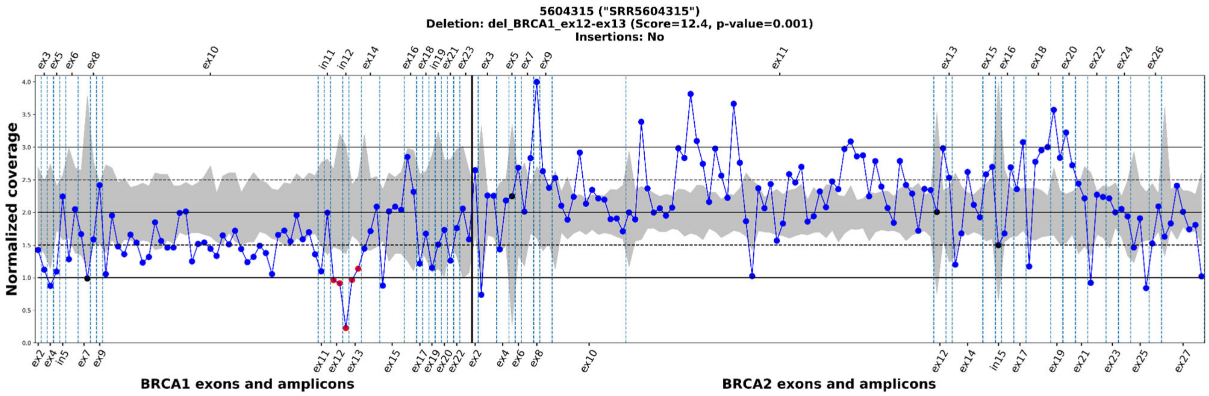

Figure S31. Ratio plots for SRR5604315. All target regions potentially affected by CNV are highlighted in red, but the final result is written in the figure top. Here it is *BRCA1* exons 12–13 deletion.
